# Neural Control of Tissue Perfusion: Emerging Evidence and Beyond

**DOI:** 10.1007/s11906-026-01378-3

**Published:** 2026-06-30

**Authors:** Eduardo Colombari, Gustavo Rodrigues Pedrino, Pedro Lourenço Katayama, Daniel Breseghello Zoccal, Michelle Mendanha Mendonça, Debora Simões Almeida Colombari, Carlos Henrique Xavier

**Affiliations:** 1 Department of Physiology and Pathology, School of Dentistry Araraquara, São Paulo State University (UNESP), Araraquara, SP Brazil; 2 Department of Physiological Science, Institute of Biological Sciences, Federal University of Goiás. Goiânia, Goiânia – GO, Brazil

**Keywords:** Neural control of circulation, Brainstem cardiovascular regulation, Vasomotor control, Cardiovascular diseases, Tissue perfusion

## Abstract

**Purpose of Review:**

Tissue perfusion is tightly regulated by central neural circuits that integrate autonomic and sensory inputs to match blood flow to tissue metabolic demand. This review synthesizes current knowledge on the central mechanisms governing tissue perfusion in both physiological and pathological states, with emphasis on the organization of brain networks involved in cardiovascular control.

**Recent Findings:**

Recent advances reveal that tissue perfusion is modulated not only by classical reflex pathways, but also by humoral, metabolic, immune, and neuromodulatory systems. Key brain regions, particularly within the brainstem, coordinate sympathetic and parasympathetic outflows through complex and state-dependent interactions that dynamically regulate vasomotion and cardiac function. Emerging evidence also demonstrates that maladaptive remodeling of these central networks contributes to vascular dysfunction in disorders such as hypertension and heart failure, promoting tissue hypoxia and end-organ damage.

**Summary:**

Central neural mechanisms play a pivotal role in the regulation of tissue perfusion under both healthy and diseased conditions. A comprehensive understanding of the neural circuits and signaling pathways involved in perfusion control may support the development of targeted therapeutic strategies aimed at restoring vascular homeostasis and improving outcomes in cardiocirculatory and neurovascular diseases.

##  The Blood Pressure and the Central Mechanisms Regulating Hemodynamics

Blood pressure (BP) is a fundamental physiological variable representing the force exerted by circulating blood against the walls of blood vessels [1]. Its principal determinants are cardiac output (CO) — the volume the heart ejects per unit time (per minute) — and the systemic vascular resistance (SVR) that the peripheral vasculature imposes on flow perfusing tissues [2]. These parameters interact continuously: variations in CO or SVR produce immediate changes in BP. The organism relies on an elaborate, multilayered control system to keep BP within a narrow healthy window, ensuring adequate organ perfusion while avoiding vascular damage [3]. The protagonists of rapid and short-term regulation of blood pressure are the neural reflexes [4–6], as they act as a negative feedback loop that constantly monitors arterial pressure, blood volume and chemical composition through specialized sensitive nerve endings called receptors, located primarily in the carotid sinus, aortic arch, heart right atria and ventricles [7].

Baroreceptors are stretch-sensitive sensors rapidly responding to blood pressure rises, increasing their firing rate that are afferently sent to the brainstem. The brainstem, specifically the nucleus tractus solitarius (NTS) in the medulla, processes this information and orchestrates a response by adjusting the activity of the autonomic (sympathetic or parasympathetic) premotor nuclei [5]. An increase in BP leads to an increase in parasympathetic (vagal) outflow to the heart, slowing heart rate, and a simultaneous decrease in sympathetic outflow, which reduces myocardial contractility and causes vasodilation of arterioles and veins, thereby decreasing SVR and adjusting CO [5, 8]. Conversely, a decrease in BP reduces baroreceptor firing, triggering increased sympathetic activity and decreased parasympathetic activity concomitantly, which results in increased heart rate, cardiac contractility, and vasoconstriction, raising BP back to normal levels and keeping tissue perfusion [3, 5, 7]. This rapid, beat-by-beat adjustment is essential for maintaining hemodynamic stability during everyday activities, like changes in posture [5, 9].

While the baroreflex is critical for short-term buffering in response to acute pressure increases, it undergoes a process of “resetting” in chronic conditions like hypertension [10], where it adapts to work at a higher pressure setpoint [3, 11–15]. This resetting means the baroreflex reduces the effectiveness while correcting sustained elevated BP, shifting the long-term control authority to renal and hormonal systems [16]. Long-term regulation of blood pressure involves slower-acting systems, a complementary mechanism whose primary effectors are the kidneys and hormonal cascades such as the well-studied renin-angiotensin-aldosterone system (RAAS), whose components modulate vasomotion and tissue perfusion through direct effects and by interplaying with the aforesaid short-term mechanisms. The contribution of humoral system to reflex resetting has been confirmed recently: AngII-dependent hypertension does not require an increase in the absolute level of sympathetic outflow but, rather, an increase in sympathetic outflow relative to the elevated MAP. This alone supports the neural contribution to the maintenance of AngII-dependent hypertension. The mechanism responsible for the apparent increase in sympathetic outflow, relative to pressure, is thought to involve a resetting of the arterial baroreflex to higher pressures [17].

The baroreflex’s sensitivity (BRS), obtained from the relationship between changes in arterial BP and corresponding changes in heart rate, is a critical indicator of cardiovascular health and has significant prognostic value [18]. Impairment of BRS is associated with various cardiovascular conditions, including hypertension and heart failure [18, 19]. Given the global prevalence and significant health impact of hypertension, which remains a leading cause of noncommunicable disease deaths worldwide [1], the continued study of baroreflex pathways and the mechanisms involved become necessary. Understanding these intricate systems can lead to the development of novel therapeutic strategies, such as drug design and electrical carotid sinus stimulation [20, 21]. Research in this area also delves into the subcellular operation of baroreceptors, exploring the roles of mechanosensitive ion channels like ASIC2 and TRPV1 in mechano-electrical transduction [7, 22], and investigates genetic influences on BRS [23], offering avenues for more targeted interventions.

Not only reflex mechanisms responding to pressure changes matter to cardiocirculatory control. The chemical composition of blood is another vital aspect to be sampled. Circulating oxygen, carbon dioxide, osmolarity and the fluid acidity levels are important variables to be finely controlled, whose fine tuning involves tissue perfusion adjustments and respiratory (tidal volume) responses [24]. The chemosensory structures placed bilaterally at the carotid body (peripheral) and at ventral interpyramidal brainstem (central) are the chemoreceptors able to respond to these aforesaid chemical variations, consequently producing autonomically mediated perfusion adjustments that are concomitant to respiratory and behavioral responses aimed at reaching greater oxygen availability and enhancing CO2 exhalation [25–27].

Besides blood pressure and its chemical variations, fluid volume distending heart walls should account either to cardiac intrinsic mechanisms regulating inotropy or to the reflex adjustments of vasomotion and peripheral resistance resulting in a CO workload that is adequate to ensure the perfusion of peripheral tissues. The cardiopulmonary reflex, also known as the Bezold–Jarisch reflex, is a powerful inhibitory cardiovascular response triggered by the activation of sensory receptors located at the cardiac walls that respond to mechanical stretch [28, 29] and chemical stimuli such as serotonin, bradykinin, prostaglandins, or certain pharmacological agents [30]. When stimulated, vagal afferent fibers conduct signals to NTS, leading to enhanced parasympathetic outflow and withdrawal of sympathetic tone. The canonic triad associated with this reflex consists of bradycardia, hypotension, and peripheral vasodilation. Functionally, the reflex is thought to serve as a protective role by reducing cardiac workload during conditions of ventricular overactivation or ischemia [29]. It can be elicited experimentally by injecting serotoninergic agonists such as phenylbiguanide, and clinically, it has been implicated in situations including inferior myocardial infarction, spinal anesthesia, hemorrhage, and severe hypovolemia [28, 30]. Although traditionally described as a cardiac reflex, its integrated effects on heart rate, vascular resistance, and autonomic balance highlight its broader role in short-term cardiovascular regulation. Understanding the Bezold–Jarisch reflex is particularly relevant in autonomic physiology, anesthesia, and critical care medicine [28, 29], where abrupt vagally mediated cardiovascular depression may have significant hemodynamic consequences.

The present review is therefore aimed at shedding light on circulatory reflex components controlling perfusion and deepens the understanding on their role in health and disease.

## Baroreflex Dysfunction Impacts Cardiovascular Health

Baroreflex dysfunction significantly impairs cardiovascular health by disrupting the body’s ability to maintain blood pressure homeostasis, leading to a cascade of adverse effects. The system is crucial for the short-term regulation of BP and cardiovascular variability [4, 5]. It acts as a negative feedback loop, sensing changes in arterial pressure via baroreceptors located in the carotid sinus and aortic arch and subsequently adjusting cardiac chronotropism and inotropism and vascular resistance through autonomic nervous system modulation. When this intricate system is dysfunctional, its ability to buffer acute BP fluctuations is compromised, leading to instability and increased cardiovascular risk [2, 4, 13, 15, 18, 31].

One of the primary impacts of baroreflex dysfunction is the development and exacerbation of hypertension. In chronic hypertension, the baroreflex can “reset” to defend a higher BP setpoint, making it less effective at correcting sustained elevated pressures [14, 15]. However, profound baroreflex failure, characterized by bilateral destruction of normal BP-sensing mechanisms, can lead to severe, volatile hypertension with systolic readings reaching up to 300 mmHg, significantly increasing cardiovascular morbidity [13]. This highlights the critical role of an intact baroreflex in preventing extreme BP fluctuations.

Heart failure (HF) is another major condition profoundly affected by baroreflex dysfunction. Impaired reflexes are strongly associated with increased mortality in patients with chronic heart failure [32, 33]. In HF, there is often ventricular-vascular uncoupling, a state where the efficient energy transfer from the heart to the systemic vasculature is disrupted. This uncoupling is exacerbated by autonomic alterations, including baroreflex-induced sympathoexcitation, which is a hallmark of HF, occurring both at rest and during exercise [2]. The elevated plasma angiotensin II (Ang II) levels frequently observed in HF contribute to arterial baroreflex dysfunction by affecting voltage-gated sodium currents in aortic baroreceptor neurons, further blunting the reflex’s effectiveness [34]. Furthermore, studies in spontaneously hypertensive rats (SHR), a model of neurogenic hypertension that mimics human clinical manifestations, show that cardiac baroreflex blunting can occur very early, even in eight-week-old animals, preceding overt disease progression, suggesting its involvement in the pathogenesis of hypertension and subsequently heart failure [19].

The prognostic value of baroreflex sensitivity (BRS) underscores its importance. A reduced BRS is a strong predictor of adverse cardiovascular outcomes across various conditions [18]. Factors like aging also contribute to baroreflex dysfunction, as the effectiveness of baroreflexes in modifying cardiac period/heart rate and vascular resistance to maintain BP homeostasis diminishes with age [5]. The study of baroreflex dysfunction is therefore critical for understanding the pathophysiology of major cardiovascular diseases and for developing therapeutic interventions. For instance, electrical carotid sinus stimulation, which aims to activate baroreflex afferent activity, has emerged as a promising approach for managing treatment-resistant arterial hypertension [20, 21]. These devices work by engaging baroreflex mechanisms to govern efferent sympathetic and parasympathetic activity, thereby contributing to long-term blood pressure control [20].

Like the studies reporting medullary Imidazoline and alpha-2 adrenergic receptors, expressed in baroreflex circuitries, as targets for centrally acting antihypertensives [35, 36], the research also continues to elucidate the molecular mechanisms underlying baroreceptor function. As a recent example, the synergistic roles of acid-sensing ion channel 2 (ASIC2) [37] and transient receptor potential vanilloid subfamily member 1 (TRPV1) in mechano-electrical transduction within arterial baroreceptors may drive further advances [38]. Continued investigation into these pathways provides avenues for novel pharmacological targets and more effective strategies to restore baroreflex function and improve cardiovascular health. The study of central pathways controlling baroreflex may help deepen the knowledge required to pave ways for further advances in the management of cardiovascular diseases relying on reflex dysfunctions.

### The Medullary Pathways Controlling Baroreflex

**Fig. 1:**
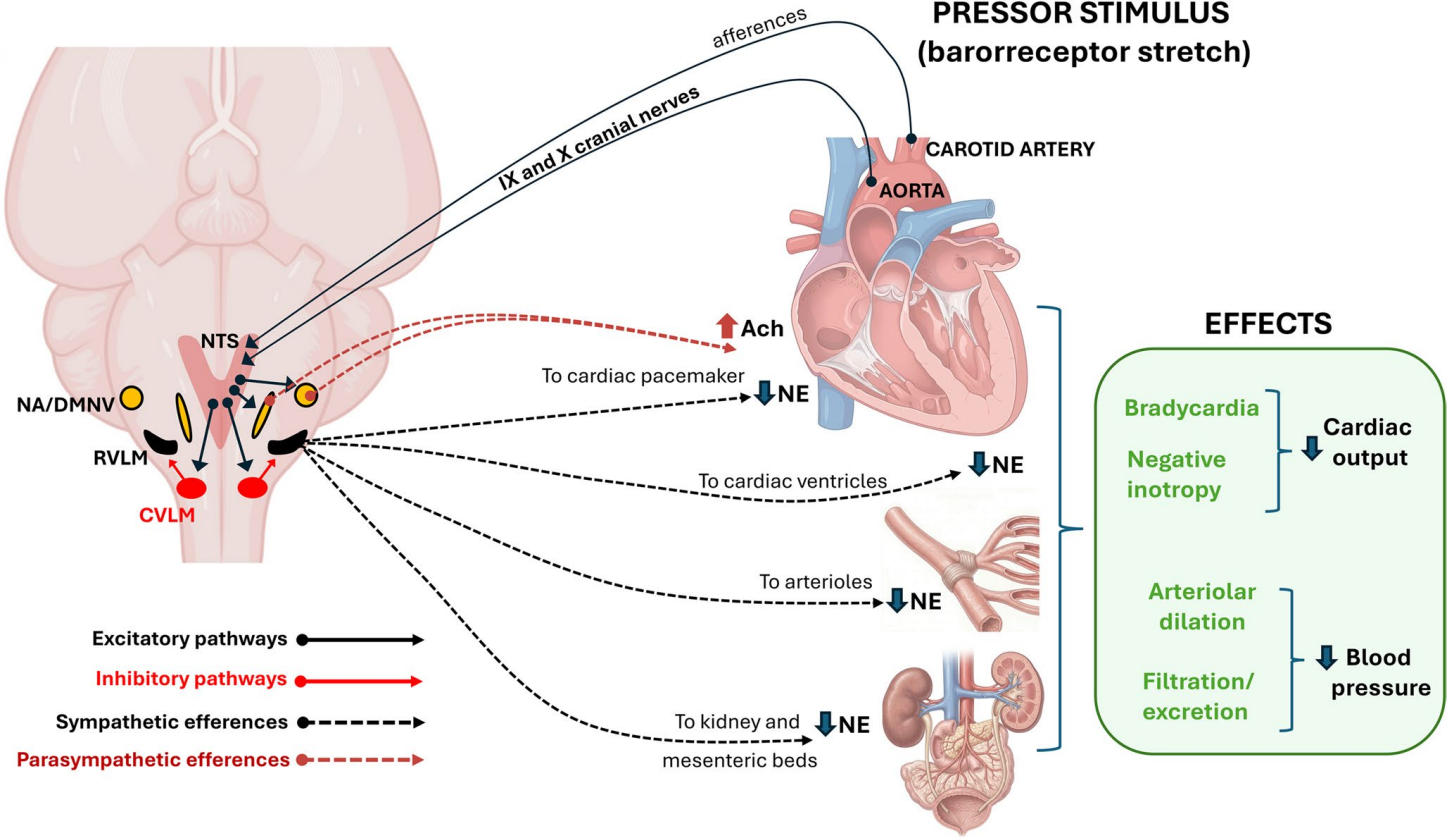
Medullary baroreflex circuitry. NA: Nucleus ambiguus. DMNV: Dorsal motor nucleus of the vagus. NTS: Nucleus tractus solitarii. CVLM: Caudal ventrolateral medulla. RVLM: Rostral ventrolateral medulla. Ach: Acetylcholine: NE: Norepinephrine

The medullary baroreflex circuitry is a critical neural pathway responsible for the beat-by-beat regulation of arterial blood pressure (BP). This intricate system operates as a negative feedback loop, integrating signals from peripheral sensors and generating appropriate autonomic responses to maintain cardiovascular homeostasis. The circuitry can be conceptualized by its key components: pressure/distension sensors, afferent pathways, central processing units, and efferent pathways [5, 39]. Figure 1 is a schematic representation of baroreflex medullary circuitry.

The neural signals generated by the pressure activation of baroreceptors are transmitted to the central nervous system via afferent nerve fibers. Baroreceptors in the carotid sinus send impulses along the carotid sinus nerve, a branch of the glossopharyngeal nerve (cranial nerve IX). Aortic arch baroreceptors transmit signals via the aortic nerve, a branch of the vagus nerve (cranial nerve X). Both sets of afferent fibers primarily terminate in the (NTS) in the caudal medulla oblongata. The NTS may be considered an “entrance gate” as it serves as the initial and crucial processing center for baroreceptor input within the brainstem [4]. Within the medulla, the NTS plays a pivotal role in integrating baroreceptor signals and orchestrating the appropriate autonomic responses [4, 40]. From the NTS, pathways extend to various other medullary nuclei that collectively regulate sympathetic and parasympathetic outflow [4]. Key medullary nuclei involved in baroreflex control include:

- **Nucleus Tractus Solitarius (NTS)**: As the primary site for baroreceptor afferent input, the NTS integrates signals related to arterial pressure changes. An increase in BP enhances baroreceptor firing, leading to increased NTS activity. This NTS activation recruits neurons projecting to subsequent inhibitory and excitatory pathways to modulate autonomic outflow.
- **Vagal Nuclei**: The NTS sends excitatory projections to the nucleus ambiguus (NA) and the dorsal motor nucleus of the vagus (DMNV), which are the main sources of parasympathetic preganglionic neurons. Activation of these vagal nuclei leads to increased parasympathetic.
- **Caudal Ventrolateral Medulla (CVLM)**: The NTS sends excitatory glutamatergic projections to the CVLM. The CVLM neurons produce the inhibitory neurotransmitter GABA as the metabolic output, whose release in synaptic cleft reaching rostral ventrolateral medulla (RVLM) neurons, reduce sympathetic outflow to heart and vessels.
- **Rostral Ventrolateral Medulla (RVLM)**: Since the pioneering studies led by Feldberg and Guertzenstein, ventral medullary surface was found as the main site controlling vasomotion [41]. The RVLM is considered the primary vasomotor center, containing sympathetic premotor neurons with tonic firing rate that provide excitatory input to sympathetic preganglionic neurons in the intermediolateral cell column of the spinal cord. When baroreceptor activity increases (due to elevated BP), NTS activates CVLM, which in turn inhibits RVLM activity. This inhibition reduces sympathetic outflow, leading to vasodilation and decreased heart rate, thus lowering BP. Conversely, a decrease in baroreceptor activity disinhibits the RVLM, increasing sympathetic tone and raising BP.

This intricate medullary network ensures the dynamic regulation of BP by balancing sympathetic and parasympathetic activity. Dysfunction in these medullary pathways can lead to impaired baroreflex sensitivity and increased blood pressure variability, contributing to conditions like hypertension and heart failure [2, 18, 34]. For example, in chronic heart failure, autonomic alterations, including increased sympathoexcitation, are mediated through central pathways influenced by the baroreflex [2, 42]. The integrity of these medullary structures is thus fundamental for maintaining cardiovascular homeostasis [4].

### The Neurotransmitters Involved in Medullary Baroreflex Circuitry

The medullary circuitry regulating baroreflex is a neurotransmitter-dependent synaptic network that relies on a complex neurochemical interplay at each synaptic stage to precisely regulate arterial blood pressure [43]. This intricate network ensures rapid and coordinated physiological responses to maintain cardiovascular homeostasis [44–47].

The primary afferent fibers that originate from baroreceptors in the carotid sinus and aortic arch terminate in the caudal portion of the Nucleus Tractus Solitarius (NTS) in the medulla oblongata. Glutamate is the primary excitatory neurotransmitter released by the baroreceptor afferents onto NTS neurons, which in turn, express ionotropic glutamate receptors, including AMPA (α-amino-3-hydroxy-5-methyl-4-isoxazolepropionic acid) receptors and NMDA (N-methyl-D-aspartate) receptors, which mediate fast excitatory synaptic transmission [45]. Metabotropic glutamate receptors (mGluRs) may also play a modulatory role.

NTS neurons activated by baroreceptor input send excitatory projections to the Caudal Ventrolateral Medulla (CVLM), with glutamate as the principal excitatory neurotransmitter in this pathway [48–50]. Post-synaptic CVLM neurons express glutamatergic receptors, mediating the excitatory input from the NTS. The CVLM plays a crucial inhibitory role by projecting to the Rostral Ventrolateral Medulla (RVLM), the primary sympathetic vasomotor center [51]. CVLM neurons produce Gamma-aminobutyric acid (GABA) as the main inhibitory neurotransmitter output that reaches postsynaptic RVLM neurons [52]. Since sympathetic premotor RVLM neurons express GABA-A receptors, there is a hyperpolarization and inhibition of RVLM neuronal activity, thereby decreasing sympathetic outflow [53–55].

As a sympathetic premotor region, RVLM neurons send direct glutamatergic projections to the spinal intermediolateral column, which connects with cell bodies of preganglionic neurons whose excitation increases noradrenergic influences on target organs such as heart, arteries, kidneys and others [55]. Conversely, an increase in GABAergic-mediated inhibition of RVLM neurons reduces noradrenaline release by post-ganglionic neurons within the synaptic cleft at autonomic synapses with target organs [56]. Bradycardia and reductions in vasomotor tones are the effects expected from baroreceptor activation by a hypertensive stimulus when sampling HR and BP [57–60].

Besides recruiting NTS-CVLM-RVLM circuitry to produce sympathoinhibition, the additional branch from the ‘first gate’ NTS neurons is also dedicated to produce an increase in parasympathetic outflow to heart. This is produced from an excitatory glutamatergic projection connecting NTS to vagal premotor/preganglionic neurons located in the nucleus ambiguus (NA) and the dorsal motor nucleus of the vagus (DMNV) [61]. These postsynaptic parasympathetic preganglionic neurons of NA and DMNV express glutamatergic receptors [62], leading to their activation and increased parasympathetic outflow to the heart [63, 64], effectively mediated by cholinergic release on cardiomyocytes, producing negative chronotropic and inotropic effects [64, 65].

This sophisticated neurochemical architecture, involving specific neurotransmitter-receptor interactions at each synaptic relay, enables the medullary baroreflex to rapidly adjust cardiovascular parameters in response to hemodynamic fluctuations, thereby maintaining blood pressure stability.

### Supramedullary Regions Influencing Baroreflex Function

Although being primarily mediated by medullary pathways, baroreflex is also significantly modulated by various supramedullary regions that integrate with other physiological functions, including emotion, cognition, and motor activity [66–69]. These higher brain centers exert influence through descending pathways that converge on the medullary baroreflex circuitry, thereby interfering with the fine-tuning regulation of blood pressure in response to complex physiological states and environmental demands [70–72].

The cerebral cortex, including the prefrontal cortex, insular cortex, and motor cortex, provides conscious and volitional control over cardiovascular function, influencing baroreflex sensitivity. The prefrontal cortex is involved in executive functions and emotional regulation, and its descending pathways can modulate autonomic outflow, impacting baroreflex activity during anticipation or cognitive tasks [73]. The insular cortex, known for its role in interoception and visceral sensation, has strong connections with autonomic centers and can integrate sensory information with cardiovascular control, affecting baroreflex gain [74]. For example, studies suggest that insular lesions can lead to altered sympathetic responses and blood pressure instability, indicating their influence on baroreflex-mediated regulation [75, 76].

The limbic system, particularly the amygdala and hippocampus, also significantly influences baroreflex function. These regions are central to emotional processing and memory. For instance, the amygdala, involved in fear and anxiety, can send direct or indirect projections to the NTS and RVLM, leading to baroreflex inhibition and sympathetic activation during emotionally charged situations [77, 78]. This modulation can manifest rapid and transient changes in blood pressure, overriding the immediate baroreflex responses to maintain homeostasis during acute stress [79]. The hippocampus, associated with memory and spatial navigation, can also modulate cardiovascular responses, although its precise role in baroreflex control is less direct and often linked to contextual memory and stress regulation [69, 80, 81].

Another key supramedullary region is the hypothalamus, which plays a pivotal role in integrating autonomic, endocrine, and behavioral responses since seminal studies [82, 83]. Specific nuclei within the hypothalamus, such as the paraventricular nucleus (PVN), anterior (AH), dorsomedial (DMH) and posterior (PH) hypothalamus, project to the medullary cardiovascular centers, including the nucleus tractus solitarius (NTS), rostral ventrolateral medulla (RVLM), and caudal ventrolateral medulla (CVLM) [84, 85]. Others, such as PVN receive dense projections from NTS, clueing the integration of medullary inputs with autonomic, neuroendocrine and hydroelectrolytic functions of diencephalic neurons. Furthermore, PVN and DMH, for example, are involved in stress responses; their recruitment can enhance sympathetic outflow and inhibit baroreflex, leading to increased blood pressure variability and sustained hypertension [72]. PVN and DMH projections reach RVLM neurons to facilitate sympathetic activation, effectively overriding or modulating baroreflex sensitivity [86], and this allows simultaneous pressor and tachycardic responses in response to aversion [81, 87].

At a little lower topographic level, the mesencephalic periaqueductal gray (PAG) is a crucial area for integrating behavioral and autonomic responses to threats, stress, and pain, also modulates baroreflex function [88] through direct projections [89]. The PAG has extensive reciprocal connections with the hypothalamus [90] and medullary cardiovascular nuclei [91], allowing it to fine-tune cardiovascular responses during fight-or-flight situations [89]. Activation of specific columns within the PAG can elicit distinct cardiovascular profiles, ranging from pressor responses with baroreflex inhibition to depressor responses, illustrating its diverse modulatory capacity.

The cerebellum, traditionally known for motor coordination, has also been implicated in cardiovascular regulation. Emerging evidence suggests that the cerebellum can influence autonomic function, including baroreflex control, possibly through its projections to brainstem nuclei involved in cardiovascular regulation [92]. While its direct impact on baroreflex sensitivity is still under investigation, it is thought to contribute to the overall stability of cardiovascular responses, especially during physical activity.

These supramedullary influences are crucial for understanding the dynamic nature of blood pressure regulation, particularly in conditions where the baroreflex might be blunted or reset, such as chronic hypertension and heart failure. For instance, chronic heart failure is associated with increased sympathoexcitation, which is mediated, in part, by central pathways influenced by altered supramedullary input, leading to ventricular-vascular uncoupling and impaired baroreflex function [2]. The ability of these higher centers to modulate the medullary baroreflex circuitry allows for complex adaptive responses, but their dysfunction can contribute to the pathophysiology of various cardiovascular diseases by disrupting the delicate balance of autonomic control.

### Main Changes in Baroreflex Function and Neurotransmission in Cardiovascular Diseases Such as Hypertension and Heart Failure

In cardiovascular diseases such as hypertension and heart failure (HF), the pathophysiological outcome results from significant deleterious changes, impacting both its function and underlying neurotransmission, leading to impaired blood pressure regulation and increased cardiovascular risk [76, 93, 94].

In hypertension, the baroreflex is frequently “reset” to a higher operating pressure, meaning it continues to function but at an elevated blood pressure set point [70]. This resetting implies that the baroreceptors become accustomed to higher pressures and perceive them as normal, thus reducing their effectiveness in buffering acute increases in blood pressure. While the exact mechanisms of resetting input-output relationship are puzzling, the involvement of structural changes in the arterial wall that alter baroreceptor mechanosensitivity and of central nervous system adaptations are uncontroversial [95]. Functionally, this resetting means that a given oscillation in arterial pressure elicits a smaller proportional reflex change in heart rate or sympathetic nerve activity when compared to a regular response in healthy conditions. This blunted response contributes to increased blood pressure variability, which is a known risk factor for cardiovascular events [94].

In heart failure, baroreflex dysfunction is even more pronounced and is a major contributor to the elevated sympathetic tone that is the hallmark of the disease [96] and for this reason, blockade of central or cardiac sympathoadrenergic receptors may be a pharmacological choice to reduce the cardiovascular risk [36]. This sympathoexcitation is not adequately counteracted by the baroreflex, which is itself impaired. This phenomenon, often referred to as autonomic imbalance, contributes to progressive ventricular dysfunction and increased arrhythmogenesis [96]. Impairment of arterial baroreflex sensitivity is indeed strongly associated with increased mortality in patients with chronic heart failure (CHF) [97].

Baroreflex changes also modify the response necessary to maintain efficient energy transfer from the heart to the systemic vasculature, a concept known as ventricular-vascular uncoupling [2]. In heart failure, this coupling is disrupted due to baroreflex dysfunction, further exacerbating the disease progression. This uncoupling is influenced by arterial baroreflex-induced sympathoexcitation resulting in positive inotropy, both at rest and during exercise.

Humoral players also take place in baroreflex dysfunction: elevated plasma angiotensin II (Ang II) is a significant factor contributing to arterial baroreflex dysfunction in CHF [98]. Ang II can acutely affect voltage-gated sodium (NaV) currents in aortic baroreceptor neurons, leading to a reduced number of these channels [34]. This reduction in NaV channels impairs the excitability of baroreceptor neurons, thus blunting the arterial baroreflex response [99]. Such molecular changes at the level of the baroreceptor afferents directly impact the initial signal transduction within the reflex arc [100].

Although literature still lacks details on the neurotransmission changes, there is evidence that chronic sympathetic activation and altered humoral factors in CHF can also lead to central sensitization of cardiovascular regulatory nuclei in the medulla, such as the NTS and RVLM [101–103]. This could involve changes in the expression or sensitivity of neurotransmitter receptors (e.g., glutamatergic, GABAergic, adrenergic) within these critical medullary pathways, contributing to the overall baroreflex impairment.

Furthermore, both hypertension and heart failure exhibit significant baroreflex dysfunction, characterized by reduced sensitivity and altered set-points [104]. In hypertension, the baroreflex resets to defend a higher blood pressure [105] whereas in heart failure, there’s a profound impairment linked to chronic sympathoexcitation and specific molecular changes in baroreceptor neurons, such as those related to Ang II and voltage-gated sodium channels [34, 98]. These alterations in baroreflex function and neurotransmission contribute significantly to the pathophysiology and progression of these cardiovascular diseases.

Notwithstanding, the cardiovascular function and pathophysiology counts on other physiological reflexes. Recent advances also pose chemoreflex as a player in circulatory alterations culminating in diseases, as discussed ahead.

## Chemoreflex as a Sensor of Blood Composition Regulating Tissue Perfusion

The peripheral chemoreflex acts as a sensor of arterial blood composition, linking changes in circulating signals to adjustments in tissue perfusion, extending well beyond its classical role in oxygen sensing and ventilatory control [106–108]. At the core of this system are the carotid bodies, small paired chemosensory organs strategically positioned at the carotid bifurcation, where they are exposed to arterial blood before it enters the cerebral circulation [109, 110]. This anatomical positioning is functionally significant: by monitoring blood composition at this critical vascular junction, the carotid bodies are ideally placed to detect deviations from homeostasis and engage reflex responses before inadequate oxygen or nutrient delivery can compromise brain function [107].

More recently, it has become evident that the sensory repertoire of the carotid bodies extends beyond the detection of hypoxemia, hypercapnia, and acidosis. A growing body of experimental evidence has established that carotid body glomus cells respond to an impressive variety of circulating molecules, including glucose [111, 112], sodium chloride [113], angiotensin II [114], leptin [115], epinephrine [116], glucagon-like peptide-1 [117], insulin [118], and a range of inflammatory cytokines and lipid mediators including TNF-α, IL-1β, IL-6, and lysophosphatidic acid [119–122]. Rather than acting as a unidimensional hypoxia sensor, the carotid body, therefore, works as a sophisticated chemical surveillance system capable of integrating multiple blood-borne signals and translating them into coordinated autonomic outputs [107, 110, 123].

The cardiovascular consequences of peripheral chemoreceptor activation are primarily driven by its influence on sympathetic vasomotor tone and, to a lesser extent, parasympathetic outflow to the heart [25, 124]. When the carotid body cells are stimulated, the resulting reflex sympathoexcitation produces vasoconstriction in peripheral vascular beds, increasing total peripheral resistance and redirecting blood flow toward metabolically active organs [125, 126]. This redistribution of perfusion is not uniform across vascular territories: the sympathetic nervous system exerts differential control over distinct regional circulations, and the chemoreflex engages these circuits selectively depending on the nature and severity of the initiating stimulus [125]. Under physiological conditions, this selectivity allows the organism to generate targeted perfusion responses without compromising global cardiovascular stability. Under pathological conditions of sustained chemoreflex overactivation, however, the same mechanisms drive chronic increases in vascular resistance, impair tissue oxygen delivery, and contribute to the progression of end-organ damage [25, 127, 128].

### The Medullary Circuitry Controlling Chemoreflex

The translation of carotid body chemosensory signals into autonomic motor commands depends on a series of interconnected brain nuclei that process afferent inputs, integrate them with other ongoing physiological signals, and generate appropriate efferent outputs to sympathetic and parasympathetic preganglionic neurons [110, 129]. The initial central processing of carotid body signals occurs in the commissural nucleus tractus solitarius (cNTS), which receives the central projections of petrosal ganglion neurons conveying chemosensory information from the carotid sinus nerve [130]. The cNTS is not a simple relay station: it integrates chemoreceptor inputs with baroreceptor signals, visceral afferent information from peripheral organs, and descending modulatory inputs from hypothalamic and supramedullary regions, integrating these signals to build an overall representation of the organism’s physiological state, which in turn shapes downstream autonomic responses [110, 129, 131].

A pivotal downstream target of cNTS chemoreflex-activated neurons is the rostral ventrolateral medulla (RVLM), which contains the majority of bulbospinal presympathetic neurons responsible for maintaining sympathetic vasomotor tone [39, 132]. Direct monosynaptic projections from cNTS neurons to the RVLM constitute a major pathway through which chemoreflex signals trigger sympathoexcitation, and the recruitment of this circuit by hypoxia or circulating inflammatory stimuli such as TNF-α has been demonstrated using retrograde tracing combined with neuronal activity markers [121, 122]. The RVLM presympathetic neurons in turn project through the intermediolateral cell column (IML) of the spinal cord to regulate the activity of sympathetic postganglionic neurons innervating vascular smooth muscle, the heart, and visceral organs [39]. Clinical and experimental evidence shows that excessive activation of peripheral chemoreceptors can disrupt proper sympathetic outflow control, contributing to the development of cardiorespiratory diseases. A particularly relevant observation in this context was made by Zoccal et al. (2008) [128], who showed that sustained activation of peripheral chemoreceptors by chronic intermittent hypoxia induces neuroplastic reorganization within the ventral respiratory group and promotes abnormal coupling between expiratory neuronal activity and sympathetic discharge, demonstrating that persistent chemosensory drive can reshape the organization of medullary cardiorespiratory networks.

The caudal ventrolateral medulla (CVLM), although not a primary component of the chemoreflex pathway, critically determines the net sympathetic output. The CVLM contains a population of GABAergic neurons that receive excitatory drive from the NTS and project to the RVLM, forming the central inhibitory limb of the arterial baroreflex [39, 50, 55]. Consequently, the level of presympathetic neuron activity in the RVLM reflects the balance between excitatory inputs arising from chemoreceptor activation via the cNTS and inhibitory baroreflex signals relayed through the CVLM, consistent with well-established interactions between baroreflex and chemoreflex pathways in the control of sympathetic outflow [39, 133]. Disruptions of this interplay contribute importantly to the dysautonomia observed in cardiovascular diseases [25, 39]. Brainstem parasympathetic nuclei, including the dorsal motor nucleus of the vagus (DMV) and the nucleus ambiguus (NA), also receive input from chemoreflex-activated NTS neurons and mediate the cardiac parasympathetic component of the reflex response [134, 135]. The respiratory rhythm and pattern generator neurons of the ventral respiratory column are additionally engaged by cNTS projections during chemoreflex activation, accounting for the hypoxic ventilatory response that accompanies and integrates with the cardiovascular adjustments [136–138].

**Fig. 2:**
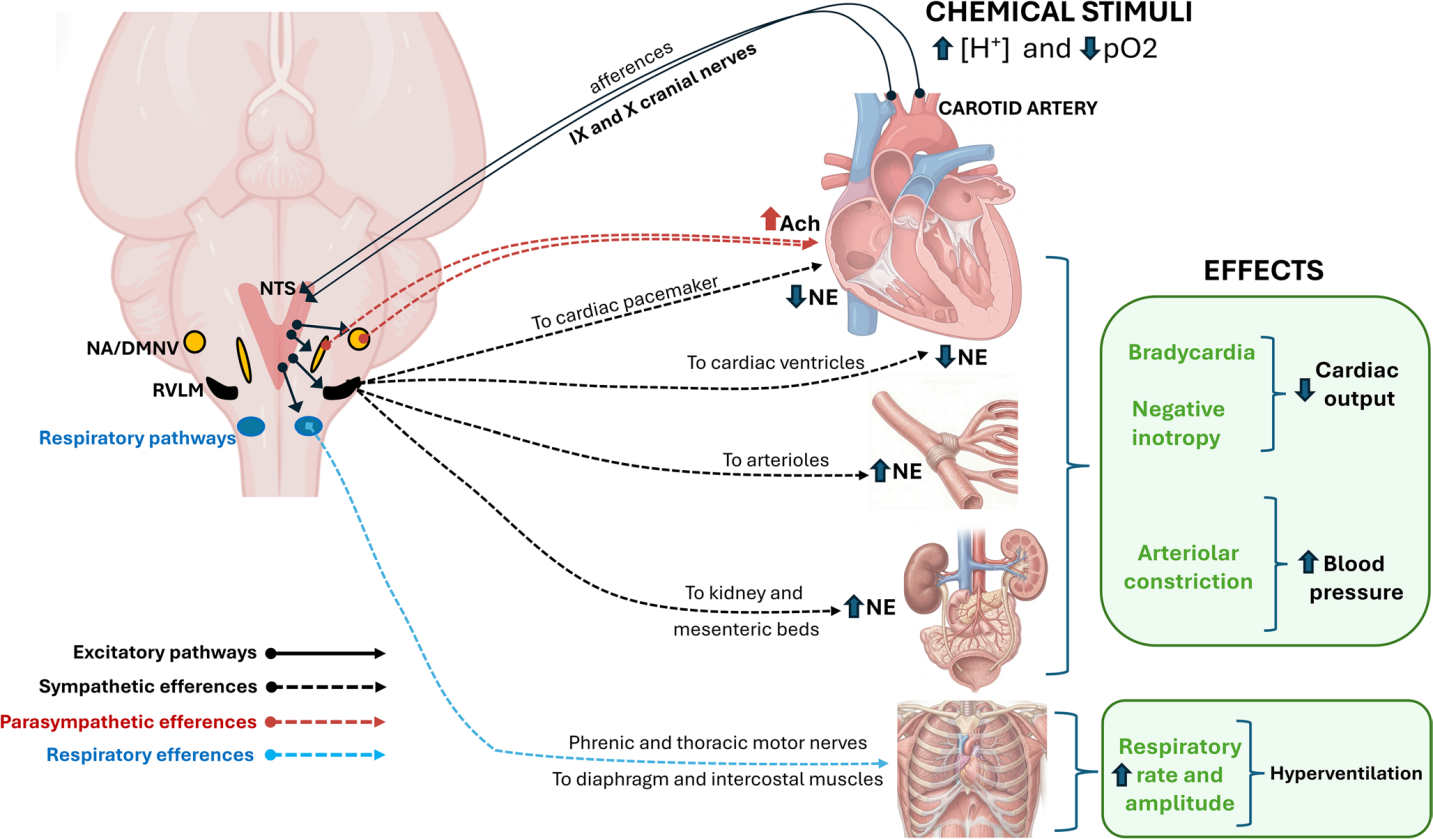
Medullary peripheral chemoreflex circuitry. NA: Nucleus ambiguus. DMNV: Dorsal motor nucleus of the vagus. NTS: Nucleus tractus solitarii. RVLM: Rostral ventrolateral medulla. Ach: Acetylcholine: NE: Norepinephrine

The functional significance of the commissural NTS as the principal entry point for chemoreceptor signals in cardiovascular regulation was highlighted by Colombari et al. (1996) [130], who showed that glutamatergic stimulation of the commissural NTS evokes robust pressor responses in conscious rats. This finding confirmed that this subdivision of the NTS is not merely a sensory relay but a functionally active node capable of driving cardiovascular responses with direct consequences for blood pressure regulation and, by extension, tissue perfusion. More recently, studies using optogenetic and chemogenetic approaches have begun to dissect the specific neuronal subtypes within the NTS that mediate chemoreflex transmission, revealing a cellular heterogeneity that likely contributes to the selectivity of autonomic responses to different blood-borne stimuli [110]. Figure 2 illustrates the medullary pathways controlling peripheral chemoreflex responses.

### The Neurotransmitters Involved in Medullary Chemoreflex Circuitry

Fast excitatory synaptic transmission at the first central synapse of the chemoreflex arc, between carotid sinus nerve terminals and cNTS neurons, is mediated predominantly by glutamate acting on both NMDA and non-NMDA ionotropic receptors [139, 140]. Pharmacological blockade of these receptors within the NTS markedly attenuates sympathetic and cardiovascular responses to carotid body stimulation, confirming that glutamatergic neurotransmission at this synapse is essential for the effective transmission of chemosensory signals to downstream autonomic circuits [141]. The glutamatergic nature of the cNTS-RVLM projection has been established through the demonstration that RVLM-projecting NTS neurons activated by chemoreflex stimulation co-express the vesicular glutamate transporter 2 (VGluT2), and that this excitatory projection drives the increase in presympathetic neuron activity underlying chemoreflex-mediated sympathoexcitation [121, 122, 129]. Additional evidence indicates that chemoreflex neurotransmission within brainstem circuits is dynamically influenced by ongoing respiratory network activity, such that respiratory phase-dependent inputs regulate the timing and gain of sympathetic neuron firing during chemoreflex activation [128, 142].

The sympathetic premotor neurons of RVLM, particularly the C1 adrenergic cell group, use glutamate as their primary fast neurotransmitter at bulbospinal synapses but additionally release a variety of cotransmitters and neuromodulators including catecholamines, substance P, neuropeptide Y, and enkephalin [39, 143]. This neurochemical complexity allows presympathetic neurons to modulate the excitability of spinal sympathetic preganglionic neurons in a context-dependent manner that goes well beyond simple fast excitatory transmission. For instance, substance P acting on NK1 receptors contributes to a slow depolarizing component that may sustain sympathetic activation during prolonged chemoreflex stimulation, whereas neuropeptide Y can exert presynaptic inhibitory effects that limit transmitter release under conditions of high-frequency firing [39].

The inhibitory neurotransmitter GABA plays an indispensable role in shaping the gain of chemoreflex-mediated sympathoexcitation by acting in both the NTS and the RVLM. At the level of the RVLM, tonic GABAergic inhibition arising from CVLM interneurons sets the baseline level of sympathetic premotor neuron activity and gates the magnitude of sympathoexcitatory responses to incoming excitatory inputs [39, 50]. The interplay between excitatory chemoreflex drive and GABAergic inhibition at this level is therefore a critical determinant of how effectively a given chemosensory stimulus is translated into peripheral sympathetic activation and vascular responses. A breakdown of this inhibitory tone, as may occur through oxidative stress-mediated impairment of GABAergic transmission in disease states, would be expected to amplify chemoreflex-mediated sympathoexcitation even without changes in the afferent limb of the reflex [25, 144].

ATP, acting on P2X purinergic receptors, represents another important transmitter both at the carotid body sensory synapse and within central chemoreflex relay nuclei. In the carotid body, ATP is the primary signal released by glomus cells upon depolarization and acts on P2 × 2/P2 × 3 receptors on afferent nerve terminals to generate action potentials that propagate toward the NTS [145, 146]. Within the NTS, purinergic signaling participates in the modulation of glutamatergic transmission from primary afferent terminals, adding yet another layer of complexity to the processing of chemosensory signals at the first central relay [146, 147]. Acetylcholine was also found to contribute to the processing of carotid body information in the cNTS through its actions on nicotinic receptors [148], further supporting the notion of complex neurotransmission interactions. Angiotensin II, acting on AT1 receptors within the NTS and RVLM, provides an important neuromodulatory influence on chemoreflex neurotransmission; its enhanced signaling within these medullary nuclei in pathological states such as heart failure has been proposed as a mechanism underlying the augmented chemoreflex gain that characterizes that condition [25, 60].

Nitric oxide (NO), synthesized by neuronal nitric oxide synthase (nNOS) within medullary neurons, exerts complex and regionally specific modulatory effects on chemoreflex neurotransmission. Within the NTS, NO appears to generally facilitate glutamatergic transmission and enhance the excitability of second-order chemoreflex neurons, while within the RVLM it may contribute to a tonic restraint of presympathetic neuron activity [144]. Reduced NO bioavailability within medullary chemoreflex circuits, as occurs because of elevated superoxide production in hypertension and heart failure, would thus be expected to enhance net sympathoexcitatory output from the RVLM, contributing to the sympathetic overactivity that marks these conditions [25, 144]. Serotonin (5-HT), released from raphe neurons projecting to the NTS and RVLM, adds yet another neuromodulatory dimension to this circuitry, with evidence suggesting that serotoninergic inputs can either facilitate or constrain chemoreflex-mediated cardiovascular responses depending on the receptor subtypes engaged and the prevailing state of the network [149, 150].

### Supramedullary Regions Influencing Chemoreflex Function: Behavioral-autonomic Interface

The cardiorespiratory responses to peripheral chemoreceptor activation are not fixed, but rather dynamically regulated depending on the arousal state, emotional context, and concurrent behavioral demands of the organism [108]. This flexibility cannot be explained solely by the properties of the carotid body or medullary relay circuits. This context-dependence reflects the continuous descending influence exerted by supramedullary structures on the gain of chemoreflex-mediated sympathoexcitation, embedding the reflex within a broader hierarchy of homeostatic and behavioral regulation [151, 152]. The clinical importance of this architecture is considerable: it provides a framework for understanding why chemoreflex sensitivity varies so dramatically across individuals and disease states, and why the same degree of carotid body activation can produce vastly different cardiovascular consequences depending on the state of higher-order circuits.

The most direct supramedullary influence on the chemoreflex responses originates from the paraventricular nucleus (PVN) of the hypothalamus. PVN neurons project monosynaptically to both the RVLM and the intermediolateral cell column of the spinal cord, allowing hypothalamic signals to amplify or restrain presympathetic neuron activity in parallel with — and independently of — the medullary reflex arc [152–154]. What makes the PVN particularly significant in this context is that it is itself activated by many of the same stimuli that recruit the peripheral chemoreflex, including hypoxia, angiotensin II, osmotic challenges, and circulating inflammatory mediators, creating a coordinated dual activation of peripheral and central components of the sympathoexcitatory response. Activated PVN neurons release vasopressin, oxytocin, corticotropin-releasing hormone, and angiotensin II at downstream synaptic targets within the RVLM and IML, each of which modifies the excitability of presympathetic and preganglionic sympathetic neurons in ways that potentiate the cardiovascular impact of concurrent chemoreflex activation [86, 153, 154]. In heart failure and chronic intermittent hypoxia, PVN activity is chronically elevated and hypothalamic angiotensin II signaling within the RVLM is markedly enhanced, constituting a supramedullary amplification mechanism that compounds the sensitization occurring simultaneously at the carotid body and medullary relay nuclei [25, 135]. The PVN, therefore, functions as an active gain-setting element whose level of activation determines how powerfully the medullary chemoreflex circuitry translates incoming afferent signals into peripheral sympathoexcitation.

The amygdala, though not a classical component of the chemoreflex circuit, is an important node linking the emotional valence of chemosensory signals to autonomic output. The central nucleus of the amygdala receives chemosensory-related input relayed via the parabrachial nucleus and projects directly to the NTS, RVLM, and hypothalamus, positioning it to modulate chemoreflex gain as a function of threat appraisal and affective state [152, 155]. Amygdala activation augments sympathoexcitatory and cardiovascular responses to chemoreceptor stimulation, and the emotional salience assigned to a given chemosensory perturbation appears to scale the autonomic response it generates [39, 155]. This circuit may be particularly relevant in panic disorder, where hypercapnic challenges elicit cardiovascular responses far exceeding those predicted by the degree of blood gas perturbation, and where structural and functional amygdala alterations have been documented [156]. The amygdala thus constitutes a site at which affective state is translated directly into modulation of reflex cardiovascular gain.

The periaqueductal gray (PAG) of the midbrain integrates limbic-hypothalamic inputs with brainstem autonomic output, coordinating autonomic responses with behavioral defense reactions across a range of physiological contexts. Receiving convergent inputs from the amygdala, hypothalamus, insular cortex, and prefrontal regions, and projecting to the NTS, RVLM, and nucleus ambiguus, the PAG forms part of the descending pathway through which behavioral state modulates chemoreflex-mediated cardiovascular responses [152, 157, 158]. Its columnar organization is functionally relevant here: the dorsal and lateral columns generate sympathoexcitatory cardiovascular activation associated with active defense, while the ventrolateral PAG mediates hypotensive, bradycardic responses associated with passive coping strategies [158]. Chemoreflex inputs to the PAG may differentially recruit these columns depending on stimulus intensity and behavioral context, such that the same chemosensory stimulus produces markedly different cardiovascular outcomes depending on the concurrent state of PAG output channels.

Ascending from the NTS, the parabrachial nucleus of the dorsolateral pons relays chemoreceptor-derived signals to the hypothalamus, thalamus, amygdala, and insular cortex [159, 160]. This ascending NTS–PBN–forebrain pathway serves functions distinct from the descending modulatory circuits described above: rather than setting reflex gain, it communicates the content of chemosensory signals to structures involved in conscious interoceptive awareness, affect, and motivated behavior, generating the subjective experience of dyspnea and driving the behavioral adaptations that accompany blood gas perturbations [159]. Crucially, engagement of the amygdala and hypothalamus via this ascending relay closes a feedback loop in which the affective processing of chemosensory information in turn modulates descending influences on reflex gain, linking perception to reflex calibration. In conditions of chronic chemosensory activation, such as obstructive sleep apnea and heart failure, persistent engagement of this pathway may contribute to anxiety, fatigue, and reduced exercise tolerance that accompany these diseases, and these affective disturbances themselves may feedback to amplify sympathetic drive through amygdalar and hypothalamic mechanisms.

The insular cortex is the primary cortical substrate for interoceptive representation of visceral and chemosensory signals. Receiving chemoreceptor-related input relayed via the NTS, PBN, and thalamus, it constructs a high-order representation of the body’s homeostatic state by integrating chemosensory information with signals from the cardiovascular, respiratory, and gastrointestinal systems [161]. Descending projections from the insula to the amygdala, hypothalamus, and brainstem autonomic nuclei provide a mechanism through which this cortical interoceptive processing shapes autonomic output and modulates chemoreflex gain [72, 162]. Structural insular changes — including gray matter loss in regions involved in autonomic regulation — have been documented in obstructive sleep apnea and hypertension, both conditions defined by heightened chemoreflex sensitivity [163]. Whether insular damage is a cause or a consequence of chemoreflex dysregulation remains unresolved, but the anatomical connections are consistent with a role for impaired cortical interoceptive processing in the maladaptive amplification of chemoreflex-mediated sympathoexcitation in cardiovascular disease.

The medial prefrontal cortex and anterior cingulate cortex, through their descending connections with the amygdala, PAG, and hypothalamus, normally exert tonic inhibitory control over autonomic arousal responses [164, 165], and this influence likely extends to the gain of chemoreflex-mediated sympathoexcitation given the direct prefrontal projections to the amygdala, PAG, and brainstem autonomic nuclei engaged by the reflex. This inhibitory tone is attenuated by acute stress, sleep deprivation, and chronic disease — conditions that overlap substantially with the cardiovascular disease populations in which chemoreflex overdrive is most pronounced. Under these circumstances, reduced prefrontal inhibition would be expected to disinhibit amygdalar and hypothalamic outputs, further amplifying chemoreflex-mediated sympathoexcitation through the descending pathways described above. That psychological stress, sleep disruption, and affective disorders are consistently associated with elevated sympathetic activity and worsened cardiovascular outcomes is at least partly consistent with this mechanism, suggesting that the behavioral and autonomic dimensions of chemoreflex dysregulation are difficult to dissociate in the clinical setting.

Collectively, this supramedullary architecture reveals that chemoreflex gain is not a fixed property of the peripheral chemoreceptor or the medullary relay but a dynamically regulated parameter shaped by descending hypothalamic, limbic, and cortical influences. The pathological amplification of chemoreflex sensitivity in cardiovascular disease likely reflects dysregulation at multiple levels of this hierarchy simultaneously — at the carotid body, within medullary relay circuits, and within the supramedullary network that ordinarily keeps reflex output calibrated to the organism’s actual physiological demands.

### Main Changes in Chemoreflex Function and Neurotransmission in Cardiovascular Diseases

The augmentation of peripheral chemoreflex sensitivity is a consistent finding across a broad spectrum of cardiovascular diseases, and its contribution to the sustained sympathetic overactivation, impaired baroreflex function, and adverse outcomes that characterize these conditions has become increasingly recognized [25, 127]. Rather than representing a consequence, enhanced chemoreflex drive in disease appears to be causally linked to the progression of autonomic dysfunction through a self-reinforcing cycle in which heightened chemosensory gain amplifies sympathetic activation, which in turn worsens the hemodynamic and neural derangements that further sensitize the carotid body [25, 166].

In systemic hypertension, convergent evidence from experimental and clinical investigations has established that tonic carotid body activity is elevated and that the reflex sympathoexcitatory response to acute hypoxia is disproportionately amplified compared to normotensive controls [167, 168]. Studies in the spontaneously hypertensive rat (SHR), an experimental model for essential hypertension, have been particularly informative, demonstrating that bilateral carotid body ablation or carotid sinus denervation substantially reduces resting sympathetic nerve activity and lowers arterial blood pressure, implicating chronic chemoreflex overdrive as a causally important contributor to the maintenance of elevated blood pressure in this genetic model of hypertension [127, 167]. Importantly, similar mechanisms have been demonstrated in renovascular hypertension. In the two-kidney, one-clip (2K1C) model, carotid body input contributes to the maintenance of the hypertensive phenotype and associated cardiorespiratory alterations, as carotid body removal reduces arterial pressure and ventilatory drive, supporting a role for peripheral chemoreceptors in angiotensin II–dependent forms of hypertension [169]. In parallel, inhibition of the commissural nucleus tractus solitarius in this model reduces sympathetic activity and arterial pressure, indicating that central integration within the NTS is also required for the maintenance of elevated sympathetic tone in renovascular hypertension [170].

Complementary clinical evidence comes from microneurographic studies showing a positive correlation between carotid body chemoreflex sensitivity and muscle sympathetic nerve activity in hypertensive patients, and from unilateral carotid body resection trials demonstrating reductions in blood pressure and sympathetic activity following the procedure [166, 168]. At the neurochemical level, enhanced angiotensin II signaling through AT1 receptors within the carotid body and within the NTS and RVLM, increased oxidative stress with consequent reduction in NO bioavailability, and dysregulation of purinergic signaling have all been implicated in the augmented chemoreflex gain of hypertension [25, 114, 144].

Heart failure is associated with particularly pronounced alterations in chemoreflex function, involving both peripheral and central components of the reflex arc [25, 171, 172]. The carotid body in experimental heart failure models undergoes substantial structural and neurochemical remodeling, including increased glomus cell excitability, elevated reactive oxygen species generation, enhanced angiotensin II signaling, and upregulation of pro-inflammatory cytokine receptors, collectively driving a marked increase in tonic and stimulus-evoked CSN discharge. Within the RVLM, enhanced AT1 receptor expression, increased angiotensin-converting enzyme activity, elevated superoxide production, and diminished NO availability create a neurochemical environment that amplifies the sympathoexcitatory impact of incoming chemoreflex signals, contributing to the marked elevation in resting sympathetic nerve activity that is a hallmark of the heart failure syndrome [25, 33, 42, 86]. The clinical relevance of this enhanced chemoreflex drive is underscored by the finding that carotid body denervation in an animal model of heart failure improved cardiac function and attenuated both sympathetic overactivity and disordered breathing [171], while carotid body resection in human patients with systolic heart failure produced reductions in sympathetic nerve activity and improvements in functional capacity [173].

Chronic intermittent hypoxia, the hallmark disturbance observed in obstructive sleep apnea, produces a well-characterized pattern of chemoreflex sensitization that persists well beyond the nocturnal hypoxic episodes and manifests as elevated daytime sympathetic activity, increased blood pressure variability, and heightened cardiovascular risk during wakefulness [123, 174]. At the cellular level within the carotid body, intermittent hypoxia activates reactive oxygen species signaling cascades involving heme oxygenase-2 and hydrogen sulfide that enhance glomus cell membrane excitability, while at the same time inducing local inflammatory remodeling characterized by upregulation of TNF-α, IL-1β, and IL-6 and their respective receptors within chemosensory tissue [174–176]. Zoccal et al. (2008) provided critical mechanistic insight into the central consequences of this peripheral sensitization, demonstrating in a rodent model of chronic intermittent hypoxia that the sustained elevation of carotid body afferent drive remodels medullary respiratory and sympathetic circuits, establishing an abnormal expiratory-sympathetic coupling that persists and contributes to elevated sympathetic vasomotor tone even under eucapnic normoxic conditions [128]. This demonstration of activity-dependent neuroplasticity within central chemoreflex circuits in response to intermittent hypoxia has important implications for understanding how sleep apnea transitions from an episodic nocturnal disturbance to a condition associated with sustained daytime cardiovascular risk.

More broadly, inflammatory mediators are substantially elevated in cardiovascular diseases including heart failure, hypertension, and atherosclerosis [177, 178], and the observation that such mediators can directly sensitize carotid body glomus cells — as shown by Katayama et al. (2022a, 2022b) in the context of circulating TNF-α — raises the possibility that the inflammatory burden intrinsic to these conditions itself contributes to chemoreflex sensitization, creating a pathological amplification loop linking immune activation, augmented chemosensory drive and sympathetic overactivation [121, 122] that may accelerate disease progression across multiple cardiovascular conditions.

## Cardiopulmonary Reflex As a Sensor of Heart Distention Regulating Cardiac Output and Tissue Perfusion

The Bezold-Jarisch reflex (BJR), often called cardiopulmonary reflex, is a complex neurocardiac reflex originating primarily from cardiac mechanoreceptors, particularly those located in atria and in the inferoposterior wall of the left ventricle. While historically associated with a triad of bradycardia, systemic vasodilation, and hypotension [179], its contemporary understanding extends to a broader role in cardiovascular regulation and pathophysiology. The mechanisms of BJR activation are diverse. Myocardial ischemia, particularly inferior myocardial infarction, is a potent stimulus. Reperfusion therapy following ischemia can also trigger the reflex [28, 180–182]. Other triggers include noxious chemical stimuli (e.g., veratrum alkaloids, serotonin), profound hemorrhage, spinal anesthesia, and even certain medical procedures such as cardiac catheterization or implantable cardioverter-defibrillator shocks [182].

The resulting efferent response involves a significant increase in parasympathetic activity to the heart, leading to bradycardia, and a decrease in sympathetic outflow to the peripheral vasculature, causing vasodilation and hypotension. The physiological role of the BJR is thought to be protective, primarily by reducing cardiac workload during conditions of ventricular overload or ischemia [28, 179, 182]. By decreasing heart rate and systemic vascular resistance, the reflex can potentially limit myocardial oxygen demand and prevent further myocardial damage.

However, in certain clinical contexts, an exaggerated or inappropriately activated BJR can have adverse consequences. For example, in patients undergoing spinal anesthesia – known to affect autonomic outputs reaching target organs – the BJR can contribute to significant hemodynamic instability. In patients with acute myocardial infarction, an exacerbation of BJR can lead to severe bradycardia and hypotension, potentially compromising coronary perfusion [28, 29, 181, 183]. Similarly, in these contexts, vigorous contraction of an underfilled ventricle may paradoxically trigger intense vagal activation. Understanding the mechanisms and implications of the Bezold-Jarisch reflex is crucial for managing various cardiovascular conditions and for interpreting physiological responses to clinical interventions [28, 184].

### The Medullary Circuitry Controlling Cardiopulmonary Reflex

The medullary pathways of the cardiopulmonary reflex, involve a specific neurocircuitry within the brainstem that mediates its characteristic physiological responses of bradycardia, hypotension, systemic vasodilation and apnea or hypopnea. While the full reflex arc includes peripheral sensors and efferent pathways, the medulla oblongata serves as the primary central integration and processing center for this reflex [185]. The reflex is initiated by cardiac mechanoreceptors that are sensitive to various stimuli. The information triggered from BJR receptors travels afferently through afferent unmyelinated vagal C-fibers reaching nucleus tractus solitarius (NTS) in the medulla oblongata [185]. The NTS is a critical relay station for visceral afferent information, acting as the primary central processing reflex inputs [186]. Within the NTS, the incoming signals are integrated and subsequently transmitted forward to other medullary nuclei to orchestrate the efferent responses [29, 185]. Specifically, NTS neurons activated by the BJR input send excitatory projections to two main sets of medullary regions:

1. **Vagal Nuclei**: The NTS excites parasympathetic preganglionic vagal premotor neurons located in the NA and the DMNV. As a direct result, there is an increase in the parasympathetic outflow to the heart, releasing acetylcholine at the cardiac pacemaker cells and atrial myocytes [187–189]. Cholinergic neurotransmission reaches cardiomyocytes expressing M2 muscarinic receptors, decreasing heart rate (bradycardia) and reducing inotropy [65].
2. **Inhibitory Pathway to Sympathetic Centers**: As for the baroreflex, the NTS also modulates sympathetic outflow. Activated NTS neurons project to the caudal gabaergic neurons of ventrolateral medulla (CVLM) that in turn send direct projections to rostral ventrolateral medulla (RVLM), thus producing an inhibitory effect over the tonic activity of these premotor neurons [29, 185]. Since RVLM is a protagonist of the sympathetic vasomotor tone, its inhibition results in a reduction of the excitatory drive to sympathetic preganglionic neurons in the intermediolateral cell column of the spinal cord. A direct consequence of this decreased sympathetic outflow is an expected reduction in norepinephrine release at the catecholaminergic neurovascular unit, reducing the vasomotion and causing vasodilation of arterioles and veins; at the end, systemic hypotension is the clinical manifestation expected.

**Fig. 3:**
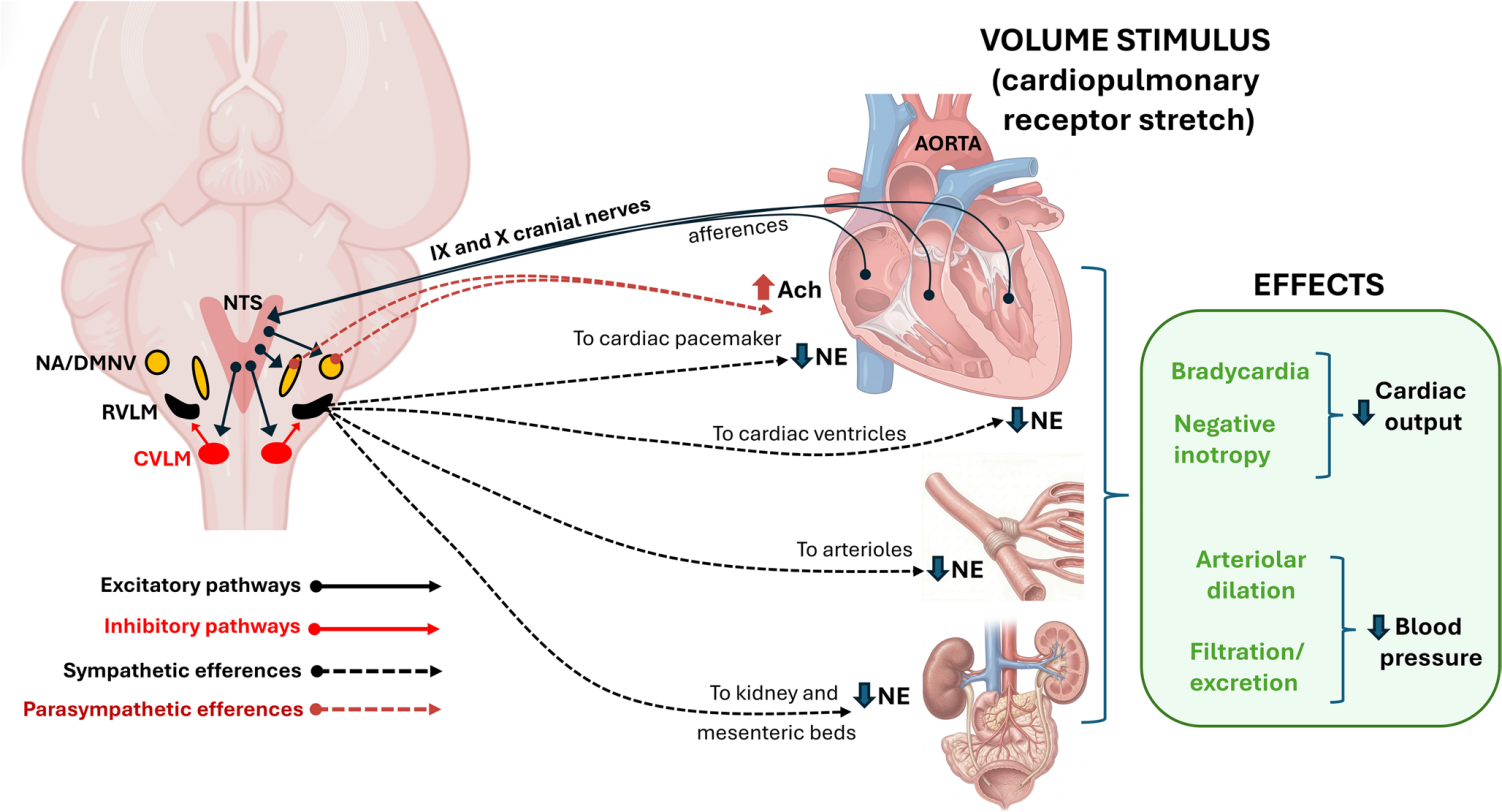
Medullary cardiopulmonary reflex circuitry. NA: Nucleus ambiguus. DMNV: Dorsal motor nucleus of the vagus. NTS: Nucleus tractus solitarii. CVLM: Caudal ventrolateral medulla. RVLM: Rostral ventrolateral medulla. Ach: Acetylcholine: NE: Norepinephrine

Therefore, the medullary pathways of the cardiopulmonary reflex effectively integrate afferent sensory information from the heart within the NTS, leading to a coordinated efferent response characterized by augmented parasympathetic activity and diminished sympathetic activity, ultimately resulting in bradycardia and hypotension [29]. These medullary interactions highlight the brainstem’s crucial role in maintaining cardiovascular homeostasis and responding to acute cardiac events. The pathways involved in BJR are shown in Fig. 3.

The respiratory – apnea or hypopnea – responses recruited from BJR are aimed at reducing the blood volume filling the heart [28], since breathing is likely to increase venous return. The same afferents reaching NTS evoked by hypervolemic heart distention also send signals to medullary respiratory areas. Although literature still lacks studies detailing the circuitries involved in the respiratory responses to BJR, candidates may be postulated based on their already described roles and on neuroanatomic possibilities of axonal projections from NTS neurons. For example, RVLM recruits ventilatory responses and ventral respiratory groups of neurons. Additionally, pre-Bötzinger complex may produce a modulation (inhibition) of inspiratory pacemaking activity, suppressing rhythmical onsets [190, 191]. Undoubtedly, further studies on central pathways coordinating ventilatory responses to the BJR and their possible behavioral integration are needed.

### The Neurotransmitters Involved in Medullary Cardiopulmonary Reflex Circuitry

The Bezold-Jarisch reflex (BJR) involves a complex interplay of neurotransmitters and their corresponding receptors, primarily at the level of the vagal afferents originating in the heart and within the medullary baroreflex circuitry in the brainstem. These neurotransmitters orchestrate the characteristic bradycardia, hypotension, and vasodilation that define the reflex [192], as follows.

#### Serotonin (5-HT) and 5-HT3 Receptors

Serotonin is a critical neurotransmitter in the initiation and modulation of the BJR. It is a potent chemical stimulus that activates the reflex when administered intravenously (5-HT or Phenylbiguanide) [186, 193, 194]. The primary receptors involved in the activation of the BJR by serotonin are the 5-HT3 receptors [195–197]. These receptors are ligand-gated ion channels found on the unmyelinated vagal afferent nerve terminals in the heart [188, 198]. Activation of these 5-HT3 receptors leads to depolarization of the vagal afferents, initiating the neural signal that travels to the brainstem [199]. Blocking 5-HT3 receptors with antagonists like ondansetron can prevent or attenuate the BJR, particularly in contexts like spinal anesthesia [200]. Cannabinoids have also been shown to affect the BJR via TRPV1 and 5-HT3 receptors [197].

#### Acetylcholine (ACh) and Muscarinic Receptors

Acetylcholine is the primary neurotransmitter mediating the efferent, parasympathetic limb of the BJR. Following the integration of afferent signals in the medulla, parasympathetic preganglionic neurons in the nucleus ambiguus and dorsal motor nucleus of the vagus are activated [150, 186]. These neurons release acetylcholine onto postganglionic neurons, which then release acetylcholine at the sinoatrial (SA) node and atrioventricular (AV) node in the heart. The action of acetylcholine on M2 muscarinic receptors on cardiac cells is responsible for the characteristic bradycardia and decreased cardiac contractility seen in the BJR [201]. Muscarinic M1 receptors in the central nervous system also play a role in the central pathway of the serotonin-induced BJR [202, 203]. Systemic administration of atropine, a muscarinic receptor antagonist, can block the cardiac response but not the sympathoinhibition to BJR activation [189, 204].

#### Glutamate and NMDA/AMPA Receptors

Glutamate serves as the primary excitatory neurotransmitter within the medullary pathways of the BJR. Vagal afferents, originating from the cardiac mechanoreceptors, terminate in the nucleus tractus solitarius (NTS) in the medulla, releasing glutamate [205]. This glutamate acts on both NMDA (N-methyl-D-aspartate) and AMPA (α-amino-3-hydroxy-5-methyl-4-isoxazolepropionic acid) receptors on NTS neurons to transmit the excitatory signals. Microinjection of NMDA antagonists into the NTS of conscious rats has been shown to block the BJR [185, 206], highlighting the critical role of these receptors in the central integration of the reflex. Furthermore, glutamatergic pathways are also involved in the excitatory projections from the NTS to the caudal ventrolateral medulla (CVLM) and to the vagal nuclei, which are crucial for mediating the reflex’s efferent responses [48].

#### Gamma-aminobutyric acid (GABA)

GABA is the main inhibitory neurotransmitter involved in the medullary processing of the BJR. NTS neurons project to the CVLM, which then sends inhibitory GABAergic projections to the rostral ventrolateral medulla (RVLM). The RVLM is responsible for maintaining sympathetic vasomotor tone. Activation of GABA-A receptors in the RVLM leads to inhibition of RVLM neuronal activity, thereby reducing sympathetic outflow and contributing to the hypotension and vasodilation observed in the BJR [50, 185, 207].

#### Other Modulators

While serotonin, acetylcholine, glutamate, and GABA are key players, other substances can modulate the BJR. For instance, the vanilloid TRPV1 receptors, activated by anandamide, are also involved in the BJR and their activation can enhance the reflex, especially during acute myocardial ischemia [208, 209]. Substance P and calcitonin gene-related peptide may also play modulatory roles in afferent signaling [195, 210], though their direct involvement in the core BJR pathway is less extensively detailed in the provided literature. The precise interplay between these various neurotransmitter systems allows for a finely tuned and adaptable response to different physiological and pathophysiological conditions that trigger the Bezold-Jarisch reflex.

### Supramedullary Regions Influencing Cardiopulmonary Reflex Function

The Bezold-Jarisch reflex (BJR) is primarily orchestrated by medullary pathways, but its function is significantly modulated by several supramedullary regions that integrate cardiovascular control with other physiological processes such as emotion, cognition, and motor activity. These higher brain centers influence the medullary baroreflex circuitry through descending pathways, allowing for a fine-tuning of blood pressure regulation in response to complex physiological states and environmental demands [211].

One critical supramedullary region is the hypothalamus, which plays a central role in integrating autonomic, endocrine, and behavioral responses. Specific nuclei within the hypothalamus, including the paraventricular nucleus (PVN), anterior hypothalamus, and posterior hypothalamus, project to key medullary cardiovascular centers such as the nucleus tractus solitarius (NTS), rostral ventrolateral medulla (RVLM), and caudal ventrolateral medulla (CVLM) [212, 213]. The PVN participation in volume reflex has been reported in health and disease, either acting through direct projections reaching medullary areas implicated in the primary reflex responses or in the hydroelectrolytic balance affecting intravascular volume and renal function [214].

Although poorly assessed, the participation of limbic system seems feasible, since brain hypoperfusion and respiratory changes may be perceived, triggering emotional components associated with distress [215]. Particularly the amygdala and hippocampus, regions that are central to emotional processing and memory, are also profoundly influence baroreflex function, which share pathways with BJR. The amygdala, involved in fear and anxiety, sends direct or indirect projections to the NTS and RVLM, leading to baroreflex inhibition and sympathetic activation during emotionally charged situations, which may play a role in the BJR [216]. This modulation can result in rapid and transient changes in perfusion, overriding immediate baroreflex responses to maintain homeostasis during acute stress. The hippocampus, associated with memory and spatial navigation, can also modulate cardiovascular responses, though its role in baroreflex control is often linked to contextual memory and stress regulation [216–219]. Periaqueductal gray at the mesencephalon is another player, since its stimulation was reported to suppress BJR-evoked bradycardia [192]. Notwithstanding, the involvement of supramedullary pathways in the organization of responses to BJR deserves exploration, since literature is scarce on a subject that may pave important ways for therapeutic interventions in situations integrating hemodynamic adjustments, respiration and behaviors like panic-attacks, apnea and others.

### Main Changes in Cardiopulmonary Reflex Function and Neurotransmission in Cardiovascular Diseases

Malfunctioning in BJR due to adaptations may compose the pathophysiology of cardiovascular diseases. In acute inferior myocardial infarction, the BJR is characteristically triggered because vagal afferent fibers are preferentially distributed in the inferior (posterior) wall of the left ventricle. Ischemic injury and local chemical mediator release in this region directly stimulate these receptors, producing sudden, severe bradycardia and hypotension [181, 220]. Studies in rodent experimental model demonstrate that the BJR response in neurogenic hypertension is attenuated compared to normotensive controls — the same pharmacological stimulus produces a smaller bradycardic and hypotensive response [221]. This blunting means the protective “brake” the BJR normally provides is weakened, contributing to the sustained sympathetic overdrive that characterizes hypertension.

Heart failure, in turn, creates multi-level disruption. The cardiopulmonary baroreflexes — the family of low-pressure reflexes to which the BJR belongs — lose their ability to restrain sympathetic outflow. In canine models of left ventricular dysfunction, cardiopulmonary baroreflex control of renal sympathetic nerve activity is markedly impaired, meaning that the normal reflex suppression of sympathetic drive to the kidneys fails [222–224]. This contributes to neurohormonal activation (sodium retention, vasoconstriction) and disease progression.

In pulmonary arterial hypertension (PAH), the BJR has been identified as a direct cause of exertional syncope. During cardiopulmonary exercise testing, vigorous right ventricular contraction against a poorly filled left ventricle can trigger the BJR, producing sudden bradycardia and hypotension during exertion [225]. This illustrates how the reflex, designed to protect the heart under conditions of relative hypovolemia, becomes pathologically activated when ventricular filling is compromised by pulmonary vascular disease.

The summarized hypotheses on core causal logic for pathophysiological changes in BJR could be: (i) at the sensor level, cardiovascular disease either over-stimulates ventricular receptors or chronically desensitizes them, causing either dangerous reflex surges or inadequate reflex protection; (ii) at the neurotransmitter level, increased platelet serotonin release in ischemia activates the reflex acutely, while chronic changes in 5-HT [194] and catecholaminergic function [222] shift the baseline sympathovagal balance toward sympathetic dominance; (iii) at the central integration level, neuroinflammation [226] and increased GABAergic tone [227] in the NTS dampen the brainstem’s ability to translate afferent signals into appropriate parasympathetic responses; (iv) at the efferent level, pre-synaptic $\:{\mathrm{Ca}}^{2+}$ dysregulation [228] and altered norepinephrine clearance [229] amplify sympathetic output, opposing the BJR’s normal inhibitory function. The result is a reflex that is either paradoxically hyperactivated in acute settings (producing hemodynamic collapse) or chronically blunted in chronic disease (removing a critical cardiovascular brake) — both pathways contributing to disease progression and adverse outcomes. Despite the consistent evidence on the alterations detected at different levels, all these important points still need to be deepened by future studies.

## Recent Advances: Intracranial Pressure

Baroreflex mechanisms that regulate cerebral perfusion are essential for the maintenance of consciousness; their significance is directly reflected in survival outcomes as central hypoperfusion results in syncope progressing to whole brain hypoxic damage. The baroreflex contributes to the control of intracranial pressure (ICP) primarily through a specialized intracranial baroreceptor mechanism [230] that ensures encephalic perfusion even when ICP rises [231]. These increases trigger a sympathetic-mediated mechanism of increase in systemic arterial pressure to counteract the reduced brain perfusion [232]. Therefore, the baroreflex does not directly regulate ICP; instead, its influence on ICP is indirect and arises from its primary role in stabilizing systemic arterial blood pressure [233]. Functionally, this high-gain negative feedback mechanism modulates autonomic efferent activity to regulate cardiac output supporting brain perfusion within a narrow physiological range [230]. Because cerebral perfusion pressure (CPP) is defined as the difference between MAP and ICP (CPP = MAP – ICP), any baroreflex-mediated change in MAP can secondarily influence ICP, particularly when cerebral autoregulation—the brain’s intrinsic ability to maintain constant cerebral blood flow across a range of perfusion pressures—is impaired [234–236].

In healthy individuals with preserved cerebral autoregulation, transient fluctuations in mean arterial pressure (MAP) are effectively buffered by reciprocal adjustments in cerebrovascular resistance, thereby maintaining relatively stable cerebral blood flow and minimizing variations in intracranial pressure (ICP). However, in pathological states such as traumatic brain injury (TBI), stroke, or malignant hypertension, autoregulatory capacity may be impaired or entirely lost. Under these conditions, baroreflex-mediated elevations in MAP can be passively transmitted to the cerebral vasculature, resulting in increased cerebral blood volume and a consequent rise in ICP, consistent with the constraints imposed by the Monro–Kellie doctrine [237, 238]. Conversely, a baroreflex-mediated drop in MAP during hypovolemia or vasodilation could reduce CPP, triggering ischemic cascades that cause cytotoxic edema and secondary ICP elevation. During hypertension, the main risk factor to stroke, ICP changes may result from peripheral humoral influences modulating brain permeability and autonomic control, thus unraveling integrative pathophysiological aspects affecting encephalic perfusion [232, 239].

Recent evidence has described a distinct “intracranial baroreflex” mechanism that appears to counterbalance the classical arterial baroreflex. This proposed pathway involves intracranial sensors capable of detecting changes in ICP and initiating sympathetic-mediated increases in MAP to preserve CPP. Experimental studies in animal models indicate that moderate, physiologically relevant elevations in ICP (up to approximately 20 mmHg) elicit proportional increases in arterial pressure, thereby contributing to the maintenance of CPP stability [233, 238]. Importantly, this intracranial baroreflex response is diminished in hypertensive states, indicating that sustained elevations in arterial pressure may compromise the brain’s capacity to preserve adequate perfusion when challenged by increases in intracranial pressure. This attenuation may contribute to worse outcomes in hypertensive patients with acute brain injury, as their compensatory capacity is diminished [238, 240].

The interplay between systemic baroreflex function and intracranial pressure (ICP) is exemplified by the Cushing reflex, a late-stage physiological response to critically elevated ICP [241]. When ICP approaches or surpasses mean arterial pressure (MAP), the resulting reduction in cerebral perfusion leads to ischemic activation of medullary cardiovascular centers. This, in turn, induces a pronounced sympathetic discharge that markedly increases MAP in an effort to restore cerebral perfusion pressure (CPP). The ensuing hypertensive state is detected by arterial baroreceptors, which counteract via enhanced parasympathetic (vagal) activity, producing bradycardia — a defining feature of the Cushing triad, alongside hypertension and disordered respiration [241]. Consequently, although the arterial baroreflex does not directly regulate ICP, it contributes to the cardiac manifestations of this critical reflex, functioning as a secondary modulator rather than a primary controller.

Clinical evidence supports the notion that baroreflex integrity influences ICP dynamics indirectly. For instance, studies in TBI patients show that impaired baroreflex sensitivity correlates with dysregulated cerebral hemodynamics and poorer prognosis [242, 243]. Additionally, non-invasive maneuvers that alter ICP, such as head-down tilt, induce changes in sympathetic activity and cerebral blood flow velocity, though these effects appear to be buffered by intact autoregulatory mechanisms over short durations [244, 245]. These findings underscore that the baroreflex contributes to ICP homeostasis not through direct neural or humoral pathways targeting CSF production, venous drainage, or intracranial compliance, but by stabilizing the upstream determinant of CPP—systemic arterial pressure.

In summary, the influence of the baroreflex on ICP is exclusively indirect, being mediated through its role in regulating resistance (vasomotion) and its functional interactions with cerebral autoregulatory mechanisms, as well as the recently proposed intracranial baroreflex. In this context, it acts as an important buffering system against systemic hemodynamic fluctuations that might otherwise disrupt intracranial homeostasis, particularly in conditions of neurological vulnerability. Nonetheless, the overall baroreflex effects depend on the functional integrity of other cerebrovascular regulatory systems.

## Conclusions and Perspectives

Neural control of tissue perfusion has emerged as a highly dynamic and integrative process, extending beyond classical reflex pathways to encompass distributed, plastic, and state-dependent networks. Recent advances highlight the interplay between central autonomic circuits, peripheral sensory feedback, humoral and local vascular mechanisms in fine-tuning regional blood flow according to metabolic demand and environmental challenges. Importantly, neuromodulatory and hormonal influences and neuroimmune interactions are now recognized as critical determinants of perfusion regulation in both physiological and pathological conditions.

Future research should prioritize cell-type-specific and circuit-level analyses, leveraging emerging technologies such as optogenetics, chemogenetics, and high-resolution imaging. A deeper understanding of these mechanisms may uncover novel therapeutic targets for cardiovascular and neurovascular disorders. Ultimately, integrating multiscale approaches will be essential to translate fundamental insights into clinical strategies aimed at restoring or optimizing tissue perfusion.

## Data Availability

No datasets were generated or analysed during the current study.
